# Characterization of all RND-type multidrug efflux transporters in *Vibrio parahaemolyticus*

**DOI:** 10.1002/mbo3.100

**Published:** 2013-07-27

**Authors:** Taira Matsuo, Koji Nakamura, Toshio Kodama, Taro Mikami, Hirotaka Hiyoshi, Tomofusa Tsuchiya, Wakano Ogawa, Teruo Kuroda

**Affiliations:** 1 Department of Microbiology, Graduate School of Medicine, Dentistry and Pharmaceutical Sciences, Okayama UniversityOkayama, Japan; 2 Pathogenic Microbes Repository Unit, International Research Center for Infectious Diseases, Research Institute for Microbial Diseases, Osaka UniversityOsaka, Japan; 3 Laboratory of Genomic Research on Pathogenic Bacteria, International Research Center for Infectious Diseases, Research Institute for Microbial Diseases, Osaka UniversityOsaka, Japan

**Keywords:** Drug resistance, multidrug efflux transporter, RND, *V. parahaemolyticus*

## Abstract

Resistance nodulation cell division (RND)-type efflux transporters play the main role in intrinsic resistance to various antimicrobial agents in many gram-negative bacteria. Here, we estimated 12 RND-type efflux transporter genes in *Vibrio parahaemolyticus*. Because VmeAB has already been characterized, we cloned the other 11 RND-type efflux transporter genes and characterized them in *Escherichia coli* KAM33 cells, a drug hypersusceptible strain. KAM33 expressing either VmeCD, VmeEF, or VmeYZ showed increased minimum inhibitory concentrations (MICs) for several antimicrobial agents. Additional four RND-type transporters were functional as efflux pumps only when co-expressed with VpoC, an outer membrane component in *V. parahaemolyticus*. Furthermore, VmeCD, VmeEF, and VmeYZ co-expressed with VpoC exhibited a broader substrate specificity and conferred higher resistance than that with TolC of *E. coli*. Deletion mutants of these transporter genes were constructed in *V. parahaemolyticus*. TM32 (*ΔvmeAB* and *ΔvmeCD*) had significantly decreased MICs for many antimicrobial agents and the number of viable cells after exposure to deoxycholate were markedly reduced. Strains in which 12 operons were all disrupted had very low MICs and much lower fluid accumulation in rabbit ileal loops. These results indicate that resistance nodulation cell division-type efflux transporters contribute not only to intrinsic resistance but also to exerting the virulence of *V. parahaemolyticus*.

## Introduction

*Vibrio parahaemolyticus* is a slightly halophilic gram-negative bacterium and is known to be a major cause of food poisoning. Outbreaks of infection with this organism have been reported in many countries (Daniels et al. 2000; Matsumoto et al. 2000; Obata et al. 2001; Lozano-Leon et al. 2003; Gonzalez-Escalona et al. 2005; McLaughlin et al. 2005; Su et al. 2005; Cabanillas-Beltran et al. 2006; Centers 2006; Sen et al. 2007). When it is ingested with food by humans, the cells of *V. parahaemolyticus* encounter various noxious compounds, such as bile, in the duodenum. Bile includes bile acids, phospholipids, cholesterol, and bilirubin. The detergent properties of bile acids are necessary for the digestion of lipids (Holt 1972), and bile acids play an important role as an antimicrobial barrier to prevent infection by pathogens. Bile acids themselves are compounds and mainly include taurocholic, glycocholic, deoxycholic, chenodeoxycholic, and cholic acids (Hofmann 1984, 1999). *V. parahaemolyticus* must resist these acids in order to infect the host. As many multidrug efflux transporters have already been reported to contribute to resistance against bile acids (Thanassi et al. 1997; Bina and Mekalanos 2001; Nishino and Yamaguchi 2001; Nishino et al. 2003; Xu et al. 2003), analyses of these transporters are important for cases of infection by *V. parahaemolyticus*.

Drug efflux from cells is one of the major mechanisms of drug resistance in bacteria. Multidrug efflux transporter genes have been estimated by whole genome sequencing to be present in many bacteria; however, only some of these genes have been characterized in detail (Nikaido 1996; Putman et al. 2000). Five major groups of drug efflux transporter families that have currently been identified are as follows: the RND (Resistance Nodulation cell Division) family, MF (major facilitator) family, MATE (Multidrug And Toxic compound Extrusion) family, SMR (small multidrug resistance) family, and ABC (ATP Binding Cassette) family. In gram-negative bacteria, such as *Escherichia coli*, *Pseudomonas aeruginosa,* and *Salmonella enterica* serovar Typhimurium, efflux transporters belonging to the RND family play an important role in drug resistance to various antimicrobial agents including bile salts (Morita et al. 2001; Nishino and Yamaguchi 2001; Nishino et al. 2006). RND-type efflux systems consist of three components: the inner membrane protein (IMP), periplasmic membrane fusion protein (MFP), and outer membrane protein (OMP). The electrochemical potential of H^+^ across cell membranes appears to be the driving force for drug efflux by RND family transporters (Zgurskaya and Nikaido 1999; Aires and Nikaido 2005). RND-type efflux systems were recently shown to be involved not only in drug resistance but also virulence in pathogenic bacteria (Buckley et al. 2006; Nishino et al. 2006; Piddock 2006; Martinez et al. 2009).

We previously characterized the VmeAB of *V. parahaemolyticus* as a member of the RND family (Matsuo et al. 2007). VmeAB conferred significantly higher minimum inhibitory concentration (MIC) values for many antimicrobial agents when expressed in *E. coli* cells. However, TM3, *vmeAB*-deficient *V. parahaemolyticus* cells, showed slight susceptibility to a few antimicrobial agents. The MIC for deoxycholate, one of the bile acids, in *vmeAB*-deficient *V. parahaemolyticus* was similar to that in the wild-type strain. The survival of TM3 in the presence of deoxycholate was slightly decreased. Other transporters existing in *V. parahaemolyticus* have been suggested to be similar to or more potent than VmeAB (Matsuo et al. 2007).

The genome sequences of *V. parahaemolyticus* RIMD2210633 have been completely determined (Makino et al. 2003). We analyzed these genome sequences and assumed that there were a large number of putative drug efflux transporter genes in the *V. parahaemolyticus* genome, with 12 putative drug efflux transporters, including VmeAB (Matsuo et al. 2007), belonging to the RND family. However, the functions of drug efflux transporters other than VmeAB remain to be elucidated. Two RND-type efflux transporters of *Vibrio cholerae*, VexAB and VexCD, have already been reported (Bina et al. 2006; Rahman et al. 2007). A double mutant of *vexAB* and *vexCD* showed a marked increase in deoxycholate susceptibility, whereas no significant change in deoxycholate susceptibility was observed in *vexAB* or *vexCD* single-deletion mutants (Bina et al. 2006). This suggests that a deficiency in one efflux pump is complemented by the other efflux pump. Therefore, some of the remaining 11 RND-type efflux transporters may be involved in the development of intrinsic resistance in *V. parahaemolyticus*.

In this study, we cloned and characterized the remaining 11 RND-type efflux transporter genes, and also constructed a series of gene deletion mutants. We showed that nine of these transporters, including the VmeAB RND-type efflux transporters, could function as multidrug efflux transporters and that VmeCD and VmeAB are central players in *V. parahaemolyticus*.

## Experimental Procedures

### Bacterial strains and growth

The bacterial strains and plasmids used in this study are listed in Table 1. *V. parahaemolyticus* RIMD2210633 was obtained from Osaka University. Bacterial cells were grown in LB medium at 37°C. Antimicrobial agents were added to the medium, as necessary. The growth of cells was monitored by measuring optical density at 650 nm.

**Table 1:** Bacterial strains and plasmids

Strain or plasmid	Relevant characteristics	Source or reference
*V. parahaemolyticus* strains		
RIMD2210633	Clinical isolate; sequenced strain	(Makino et al. 2003)
AQ3334	Clinical isolate	(Kuroda et al. 1994)
TM3	AQ3334 *ΔvmeAB*	(Matsuo et al. 2007)
TM32	AQ3334 *ΔvmeAB, ΔvmeCD*	This study
TM33	AQ3334 *ΔvmeAB, ΔvmeCD, ΔvmeEF*	This study
TM34	AQ3334 *ΔvmeAB, ΔvmeCD, ΔvmeEF, ΔvmeYZ*	This study
TM35	AQ3334 *ΔvmeAB, ΔvmeCD, ΔvmeEF, ΔvmeK, ΔvmeYZ*	This study
TM36	AQ3334 *ΔvmeAB, ΔvmeCD, ΔvmeEF, ΔvmeHI, ΔvmeK, ΔvmeYZ*	This study
TM37	AQ3334 *ΔvmeAB, ΔvmeCD, ΔvmeEF, ΔvmeHI, ΔvmeK, ΔvmeLM, ΔvmeYZ*	This study
TM38	AQ3334 *ΔvmeAB, ΔvmeCD, ΔvmeEF, ΔvmeHI, ΔvmeK, ΔvmeLM, ΔvmeUV, ΔvmeYZ*	This study
TM39	AQ3334 *ΔvmeAB, ΔvmeCD, ΔvmeEF, ΔvmeHI, ΔvmeK, ΔvmeLM, ΔvmeWX, ΔvmeUV, ΔvmeYZ*	This study
TM310	AQ3334 *ΔvmeAB, ΔvmeCD, ΔvmeEF, ΔvmeHI, ΔvmeK, ΔvmeLM, ΔvmeQ, ΔvmeWX, ΔvmeUV, ΔvmeYZ*	This study
TM311	AQ3334 *ΔvmeAB, ΔvmeCD, ΔvmeEF, ΔvmeHI, ΔvmeK, ΔvmeLM, ΔvmeQ, ΔvmeRS, ΔvmeWX, ΔvmeUV, ΔvmeYZ*	This study
TM312	AQ3334 *ΔvmeAB, ΔvmeCD, ΔvmeEF, ΔvmeHI, ΔvmeK, ΔvmeLM, ΔvmeO, ΔvmeQ, ΔvmeRS, ΔvmeWX, ΔvmeUV, ΔvmeYZ*	This study
TM4	AQ3334 *ΔvmeCD*	This study
TM5	AQ3334 *ΔvmeEF*	This study
TM6	AQ3334 *ΔvmeHI*	This study
TM7	AQ3334 *ΔvmeK*	This study
TM8	AQ3334 *ΔvmeLM*	This study
TM9	AQ3334 *ΔvmeO*	This study
TM10	AQ3334 *ΔvmeQ*	This study
TM11	AQ3334 *ΔvmeRS*	This study
TM12	AQ3334 *ΔvmeUV*	This study
TM13	AQ3334 *ΔvmeWX*	This study
TM14	AQ3334 *ΔvmeYZ*	This study
TM15	AQ3334 *ΔvpoC*	This study
TM425	AQ3334 *ΔvmeAB, ΔvmeCD, ΔvmeEF, ΔvmeHI, ΔvmeK, ΔvmeLM, ΔvmeO, ΔvmeQ, ΔvmeRS, ΔvmeWX, ΔvmeUV, ΔvmeYZ, ΔvpoC*	This study
RTM3	RIMD2210633 *ΔvmeAB*	This study
RTM4	RIMD2210633 *ΔvmeCD*	This study
RTM32	RIMD2210633 *ΔvmeAB, ΔvmeCD*	This study
RTM313	RIMD2210633 *ΔvmeAB, ΔvmeCD, ΔvmeEF, ΔvmeHI, ΔvmeJK, ΔvmeLM, ΔvmeO, ΔvmeQ, ΔvmeRS, ΔvmeWX, ΔvmeUV, ΔvmeYZ*	This study
*E. coli* strains		
KAM33	TG1 *ΔacrAB*, *ΔydhE*	(Matsuo et al. 2007)
KAM43	KAM33 *ΔtolC*	(Matsuo et al. 2007)
Plasmids		
pSTV28	Expression vector: Cm^r^; multiple cloning site in *lacZ*	
pSTV29	Expression vector: Cm^r^; multiple cloning site in *lacZ*	
pBR322	Expression vector: Amp^r^, Tet^r^	
pRHR228	*vmeAB* cloned into pSTV28, Cm^r^	(Matsuo et al. 2007)
pSVP201	*vmeCD* cloned into pSTV28, Cm^r^	this study
pSVP202	*vmeEF* cloned into pSTV29, Cm^r^	This study
pSVP204	*vmeGHI* cloned into pSTV29, Cm^r^	This study
pSVP205	*vmeJK* cloned into pSTV28, Cm^r^	This study
pSVP206	*vmeLM* cloned into pSTV28, Cm^r^	This study
pSVP207	*vmeNO-vpoM* cloned into pSTV28, Cm^r^	This study
pSVP208	*vmePQ* cloned into pSTV28, Cm^r^	This study
pSVP209	*vmeRS* cloned into pSTV29, Cm^r^	This study
pSVP210	*vmeTUV* cloned into pSTV28, Cm^r^	This study
pSVP211	*vmeWX* cloned into pSTV28, Cm^r^	This study
pSVP212	*vmeYZ* cloned into pSTV28, Cm^r^	This study
pSET2	*tolC* cloned into pSTV29, Cm^r^	(Li et al. 2008)
pSVT2	*vpoC* cloned into pSTV29, Cm^r^	This study
pBET2	*tolC* cloned into pBR322, Amp^r^	This study
pBVT3	*vpoC* cloned into pBR322, Amp^r^	(Matsuo et al. 2007)

### Construction of a plasmid library containing putative RND-type multidrug efflux transporter ORFs

Open reading frames (ORFs) assumed to be RND-type multidrug efflux transporter genes were cloned from the chromosomal DNA of *V. parahaemolyticus* RIMD2210633. ORFs were amplified without putative native promoters by polymerase chain reaction (PCR) using the pair of primers shown in Table 2. The putative outer membrane protein gene, VPA0362 (designated *vpoM*), was amplified with a membrane fusion protein gene (VPA0364, *vmeN*) and inner membrane protein gene (VPA0363, *vmeO*) because *vpoM* appears to form an operon with *vmeN* and *vmeO* under the control of a promoter upstream of *vmeN*. PCR was performed with a PTC-100™ Programmable Thermal Controller (MJ Research Inc., Quebec, Canada) using KOD -plus- DNA polymerase (TOYOBO Co., Ltd, Kita-ku, Osaka, Japan). PCR products were confirmed with 1% agarose gel electrophoresis and purified by the GENECLEAN II kit (MP Biomedicals Japan, Chuo-ku, Tokyo, Japan). DNA fragments were digested with a restriction enzyme and then ligated at the multicloning sites of pSTV28 or pSTV29 (TAKARA BIO Inc., Otsu, Shiga, Japan). All RND multidrug efflux transporter genes were ligated under the *lac* promoter of pSTV vectors. *E. coli* KAM33 competent cells were transformed with recombinant plasmids. The nucleotide sequences of ORFs in recombinant plasmids were determined by the dideoxy chain termination method, and were confirmed to match sequences in the database (http://genome.naist.jp/bacteria/vpara/).

**Table 2:** Primers used in this study

Primer	Sequence
For gene cloning	
vmeCD-F	CACCAGGATCCAATTATCAAACACTAACTTG
vmeCD-R	GAAAGGATCCTCGCCATTTAGATGGTAAAA
vmeEF-F	CAGGGGATCCAGTTTAATGACATAAGTTT
vmeEF-R	CCGAGGATCCTAGAAATATAAAAAAACGCC
vmeGHI-F	CGGCGGATCCTAATTCATCTACTTTAAATG
vmeGHI-R	CACGGAATTCTATAAAAAGCGCCTCTAACT
vmeJK-F	GAGAGGATCCAGGAGAGAATAATAAAAAGG
vmeJK-R	AGAGGGATCCAATGAGATAAACGGAAAAGT
vmeLM-F	AGGAGGATCCAAAAACAACAAGAGCATTC
vmeLM-R	TAAAGGATCCAAAAAAAGCAGCCCGAAGG
vmeNO-vpoM-F1	GGTCGGATCCTTTTGTAGCTTGCATTTAT
vmeNO-vpoM-R1	TTTTAGGCAGCTGCAGTTTTACATCGTC
vmeNO-vpoM-F2	AACCATATAAAACTGCAGCTGCCTTCCA
vmeNO-vpoM-R2	CTCTCTGCAGCCTCTTTTTAACCGATTTAA
vmePQ-F	GAGCGGATCCCATAAAGTAGGTTTATC
vmePQ-R	ATAAGGATCCAAAATCAGCGTTATTGCTCG
vmeRS-F	GGAAGGTACCGGAAAATAAGGAATTAGGAAT
vmeRS-R	TGTCGGTACCCTTTTTCATGTTGATTTCCT
vmeTUV-F	CGCTGGTACCGACTGTGTAGTAAATTTTAA
vmeTUV-R	ACGAGCATGCAAAACGAAAAAAGCCCTGA
vmeWX-F	CACAGGATCCAACGCGTAAACAATCAATCT
vmeWX-R	GTAAAGGATCCATAGTGTGTTAGATAGACG
vmeYZ-F	AATAAGGATCCATCCTGTTCCATAAAGACG
vmeYZ-R	TTCGGGATCCTTTTTGTTTTCTATCTAGG
For RT-PCR	
vmeB RT-F	CTGCGACCATTACACTGACTT
vmeB RT-R	GTGTGTAAAGTCTGGATCGTC
vmeD RT-F	GGAGCAAGCAGTAGCACAAG
vmeD RT-R	AAGCCGATGTACAGTACTGC
vmeF RT-F	CCACCACTCGTAACAGCATG
vmeF RT-R	TGTGCGGTCCATTTCAGAAG
vmeI RT-F	GTGGAAGAAGAAGTGACGTAC
vmeI RT-R	CCCGAAGTCATCAATAACCTG
vmeK RT-F	AGAACTGCGCTCAATTGAAG
vmeK RT-R	TCCTCTGAAGCAAAAGTGAC
vmeM RT-F	TCAAAGTGGAAGAGTCGATC
vmeM RT-R	CGTCTTGTCGCCATTCAAC
vmeO RT-F	ATCGCTTGCGCTCATTGTCT
vmeO RT-R	AACTCTTCTGGCGCAGCAT
vmeQ RT-F	CTGTGATTGGTAACGATGGC
vmeQ RT-R	CGTAAGACTGTTTCGGGGAA
vmeS RT-F	TTCTTCGGGGATTCGTACGT
vmeS RT-R	AGCTCGGCAAGGTCATGTT
vmeV RT-F	GAATGGTATGTCGCAGATCA
vmeV RT-R	GCGTTTGAGCTCGACATAGT
vmeW RT-F	CCAAATTGACGGTGTGGAATAT
vmeW RT-R	CGGCAACAACAATGGGTACA
vmeZ RT-F	CGAAAAGTGCTAACGATGGT
vmeZ RT-R	GTCATCATACTTGCCGTCTG
16S rRNA RT-F	ACGTTAGCGACAGAAGAAGC
16S rRNA RT-R	ACCGCTACACCTGAAATTCT

### Construction of pBET2 carrying the *tolC* of *E. coli*

A *Xba*I-*Fsp*I fragment from the pSET2 plasmid (Li et al. 2008) carrying the *tolC* gene was ligated to pBR322 digested with *Nhe*I and *Nru*I. The resulting plasmid was named pBET2. The inserted *tolC* gene without a native promoter region was located downstream of the *tet* promoter of the same orientation.

### Drug susceptibility test

The MICs of various antimicrobial agents were determined in Mueller–Hinton broth (BD Japan, Minato-ku, Tokyo, Japan) containing different drugs at various concentrations as described previously (Matsuo et al. 2007). In order for cloned genes to be expressed, 0.5 mmol/L isopropyl-β-D-thiogalactopyranoside (IPTG) was added as necessary. Cells were incubated in the test medium at 37°C for 24 h, and growth was examined by visual inspection. MIC was defined as the lowest concentration of a drug that inhibited visible growth.

### RT-PCR analysis

Total RNA was extracted from the cells of *V. parahaemolyticus* AQ3334 and RIMD2210633 grown in LB medium or LB medium containing 0.2% sodium deoxycholate at the exponential phase with the QIAGEN RNeasy Mini Kit (QIAGEN, Chuo-ku, Tokyo, Japan). Extracted total RNA was treated with RNase-free DNase (Promega KK, Chuo-ku, Tokyo, Japan) and re-purified using the same kit. Reverse transcription polymerase chain reaction (RT-PCR) was performed with the QIAGEN One-Step RT-PCR Kit (QIAGEN Inc.). PCR without a reverse transcription reaction was performed to confirm the absence of detectable DNA contamination. RT-PCR products were analyzed by 3% agarose X gel (NIPPON GENE CO., LTD., Chiyoda-ku, Tokyo, Japan) electrophoresis. Primers used for RT-PCR are described in Table 2. The amplification efficacy of each primer set was compared using chromosomal DNA as a template (data not shown).

### Ethidium efflux assay

Ethidium efflux assays were performed as described previously with a slight modification (Rahman et al. 2007). In brief, *E. coli* KAM43 cells harboring both pBVT3 (carrying *vpoC*) and pSTV28 (empty vector), and *E. coli* KAM43 cells harboring both pBVT3 and either pSVP201 (carrying *vmeCD*) or pSVP202 (carrying *vmeEF*) were grown in LB broth supplemented with 100 μg mL^−1^ of ampicillin and 20 μg mL^−1^ of chloramphenicol with 40 mmol/L potassium lactate and 0.5 mmol/L IPTG at 37°C up to the late exponential phase of growth. Cells were harvested and washed twice with modified Tanaka medium (Tanaka et al. 1967) in which sodium salts were replaced with potassium salts and supplemented with 2 mmol/L MgSO_4_. Cells were resuspended in the same buffer including 10 μmol/L ethidium bromide and 40 μmol/L carbonylcyanide *m*-chlorophenylhydrazone (CCCP) to starve energy and load ethidium, and were incubated at 37°C for 1 h. Cells were harvested and washed twice with the same buffer containing 10 μmol/L of ethidium bromide and 2 mmol/L MgSO_4_. Potassium lactate (40 mmol/L) was added for energization. The fluorescence intensity of ethidium was measured at an excitation wavelength of 500 nm and emission wavelength of 580 nm, respectively, using an F-2000 Fluorescence Spectrophotometer (Hitachi, Ltd., Chiyoda-ku, Tokyo, Japan).

*V. parahaemolyticus* cells were grown in LB broth supplemented with 40 mmol/L potassium lactate, and washed twice with Medium S (50 mmol/L Tris-HCl, 200 mmol/L NaCl, 25 mmol/L MgSO_4_, 10 mmol/L (NH_4_)_2_SO_4_, 10 mmol/L KCl, 1 mmol/L CaCl_2_, 0.33 mmol/L K_2_HPO_4_, 10 μmol/L FeSO_4_, pH 7.5). Cells were incubated in Medium S including 10 μmol/L ethidium bromide and 40 μmol/L CCCP at 37°C for 1 h. Cells were harvested and washed twice with the same buffer containing 10 μmol/L of ethidium bromide. Potassium lactate (40 mmol/L) was added for energization.

### Gene disruption by homologous recombination

Gene disruption was accomplished in *V. parahaemolyticus* as described previously (Kuroda et al. 2005; Matsuo et al. 2007). In brief, the plasmids pXAC1092, pXAC0038, pXAC0941, pXAC1178, pXAC2472, pXAC0344, pXAC0363, pXAC0471, pXAC0480, pXAC0809, pXAC0944, pXAC1190, and pXAC0425 were constructed, and each of these plasmids carried each inactivated operon *vmeAB*, *vmeCD*, *vmeEF*, *vmeGHI*, *vmeJK*, *vmeLM*, *vmeNO*, *vmePQ*, *vmeRS*, *vmeTUV*, *vmeWX, vmeYZ*, and *vpoC*, respectively. Each plasmid was introduced into *E. coli* β2155 (Herz et al. 2003). These donor strains harboring each plasmid, carrying the deleted RND type genes, and recipient strains were harvested at the early exponential phase of growth and mixed gently. The mixed cell suspension was trapped on a membrane filter (0.2 μm pore size, Toyo Roshi Kaisha, Ltd., Bunkyo-ku, Tokyo, Japan). After washing, the filter was put on an LB broth agar plate and incubated at 37°C for 3 h. Cells were suspended in LB broth and shaken at 37°C for 1 h. The cell suspension was spread onto an LB broth agar plate (2.5% agar for vibrio) containing 5 μg mL^−1^ chloramphenicol and 50 μg mL^−1^ ampicillin. Ampicillin was added to allow only *V. parahaemolyticus* cells to grow. The plate was incubated at 30°C overnight. Chloramphenicol-resistant and ampicillin-resistant candidates were picked and used for the next step. These candidates were cultured in LB broth without any antibiotics, spread onto a VDS agar plate (1% polypeptone, 0.5% yeast extract, 30 mmol/L NaCl, 55 mmol/L KCl, 10% sucrose, and 2.5% agar), and incubated at 25°C or below. When a second recombination occurred, cells were expected to become resistant to sucrose and sensitive to chloramphenicol. By amplifying the corresponding gene region with PCR, we confirmed that genes were disrupted.

### Effect of sodium deoxycholate on the survival rate of *V. parahaemolyticus* strains

*V. parahaemolyticus* cells in the exponential phase (2.72–7.37 × 10^6^ cells) were suspended in 1 mL of 0.5 mol/L NaCl-PB (0.1 mol/L phosphate buffer, pH 7.2) including 1 mg mL^−1^ of sodium deoxycholate. After incubation at 37°C for 10 min, cells diluted 10^−4^ with 0.5 mol/L NaCl-PB were spread onto an LB agar plate (2.5% agar). Colony-forming units were counted following incubation at 37°C for 12 h. Three individual experiments were performed and similar results were obtained.

### Rabbit ileal loop test

The rabbit ileal loop test was performed as described previously (Hiyoshi et al. 2010). Isogenic mutant strains of *V. parahaemolyticus* (10^9^ CFU/loop) were injected into the ligated rabbit ileal loop followed by the measurement of fluid accumulation in each loop 18 h after the injection. Fluid accumulation (FA) ratios were calculated as the amount of accumulated fluid (mL) per length (cm) of ligated rabbit small intestine. All animal experiments were performed according to the experimental protocol approved by the Ethics Review Committee for Animal Experimentation of the Research Institute for Microbial Diseases (Osaka University, Osaka, Japan).

### CAS assay

The preparation of chrome azurol S (CAS) solution and the CAS assay were performed as described previously (Schwyn and Neilands 1987). Bacterial cells were grown in LB medium to the late exponential phase of growth under aerobic conditions at 37°C. Cells were harvested and washed twice with medium SF with no FeCl_3_ in Medium S, were inoculated into the same medium containing 0.2% glucose at an optical density of 650 nm (O.D._650_) of 0.5, and were shaken at 37°C. Growth was monitored by measuring O.D._650_. A 35 μL aliquot of supernatant was mixed with 70 μL CAS assay solution. The mixture was incubated at room temperature for 2 h, and the absorbance was then measured at 630 nm.

## Results

### Search for RND-type efflux transporter genes in the *V. parahaemolyticus* RIMD2210633 genome

**Figure 1:**
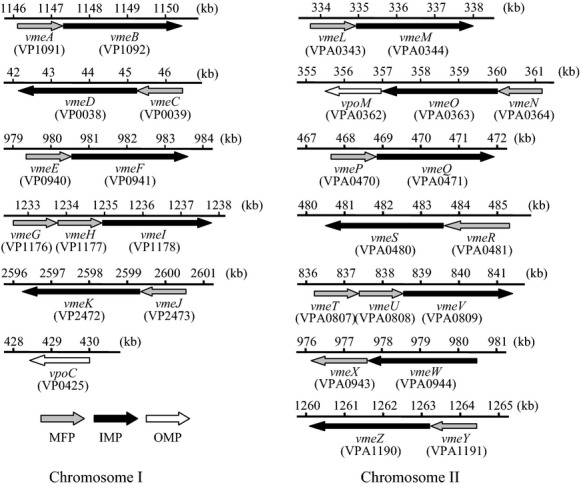
RND-type efflux transporter genes encoded in the *V. parahaemolyticus* genome. The chromosomal positions of genes coding for the putative RND-type efflux transporters, outer membrane proteins, and membrane fusion proteins are indicated by the kb in the *V. parahaemolyticus* RIMD2210633 genome. Arrows correspond to the lengths and directions of the genes.

Twelve operons for RND-type multidrug efflux transporters were estimated to be present according to the genome sequence of *V. parahaemolyticus* RIMD2210633 (http://genome.naist.jp/bacteria/vpara/). One of them, VmeAB, has already been characterized (Matsuo et al. 2007). We designated the other genes and they are shown in Figure 1. MFP genes were located upstream of each IMP gene except for *vmeW*. The MFP gene, *vmeX,* was located downstream of *vmeW*. Two consecutive MFP genes were located upstream of *vmeI* and *vmeV*. One OMP gene, designated *vpoM*, was located downstream of *vmeO*.

### Cloning of RND-type multidrug efflux transporter genes and analysis in *E. coli* cells

Among these 12 operons, only *vmeAB* has already been cloned (Matsuo et al. 2007). We cloned the other 11 operons into multicopy plasmids at the downstream position of the *lac* promoter (without a putative original promoter) to achieve their effective and definite expression in *E. coli* cells (Table 1). These recombinant plasmids were introduced into drug-hypersusceptible *E. coli* KAM33 (Matsuo et al. 2007; Rahman et al. 2007), and we then investigated whether the introduction of these genes elevated drug resistance in host cells (Table 3). Three of them, VmeCD (pSVP201), VmeEF (pSVP202), and VmeYZ (pSVP212), conferred elevated drug resistance on *E. coli* KAM33 cells. Fold increases by VmeCD, in particular, on the MICs of SDS and bile acids were very high (SDS: >512-fold and bile acids: >32-fold). VmeEF conferred the elevated MIC of ethidium bromide (twofold) only. VmeYZ conferred high resistance to SDS (>512-fold), taurocholate (>eightfold), and glycocholate (>eightfold). The other eight plasmids did not render any increased resistance to the antimicrobial agents tested.

**Table 3:** MICs of various antimicrobial agents for *E. coli* KAM33 transformants

	MIC (μg mL^−1^)			
	
Antimicrobial agent	KAM33/pSTV28 (control)	KAM33/pSVP201 (*vmeCD*)	KAM33/pSVP202 (*vmeEF*)	KAM33/pSVP212 (*vmeYZ*)
Oxacillin	1	2	1	1
Erythromycin	2	4	2	2
Norfloxacin	0.03	0.03	0.03	0.03
Novobiocin	2	2	2	2
Kanamycin	0.5	0.5	0.5	0.5
Tetracycline	0.5	0.5	0.5	0.5
Ethidium bromide	2	2	4	2
Rhodamine 6G	8	16	8	8
Acriflavine	2	2	2	2
TPPCl	4	16	4	4
Hoechst 33342	0.25	0.25	0.25	0.25
SDS	100	>51,200	100	>51,200
Cholate	4000	>32,000	4000	16,000
Deoxycholate	1000	>32,000	1000	2000
Taurocholate	4000	>32,000	4000	>32,000
Glycocholate	4000	>32,000	4000	>32,000

SDS, sodium dodecyl sulfate; TPPCl, tetraphenylphosphonium chloride.

RND-type efflux transporters have been shown to consist of three components; IMP, OMP, and MFP. No OMP gene has been identified downstream of each operon, except for *vmeNO-vpoM*. The above three RND-type efflux transporters, VmeCD, VmeEF, and VmeYZ, which conferred drug resistance on *E. coli* cells, appeared to utilize TolC as an OMP component in *E. coli*. These efflux transporters could not elevate drug-resistant levels in *E. coli* KAM43, which is a *tolC* deletion mutant of KAM33 (data not shown). We previously reported that VmeAB could utilize VpoC, which is a TolC-like outer membrane protein in *V. parahaemolyticus* that confers similar drug resistant levels to *E. coli* cells (Matsuo et al. 2007). We investigated whether the other 11 RND-type efflux transporters could utilize VpoC as an OMP component. We measured the MICs of various drugs to *E. coli* KAM43 harboring both pBVT3 carrying *vpoC* and each RND-type efflux transporter gene recombinant plasmid (Table 4, upper panel). Interestingly, additional four recombinant plasmids (pSVP204, pSVP205, pSVP206, and pSVP210) conferred drug resistance on *E. coli* cells. These results suggested that VmeGHI, VmeJK, VmeLM, and VmeTUV formed functional complexes with the VpoC of *V. parahaemolyticus*. Furthermore, VmeCD-VpoC, VmeEF-VpoC, and VmeYZ-VpoC conferred higher resistance than VmeCD-TolC, VmeEF-TolC, and VmeYZ-TolC, respectively. VmeEF-VpoC and VmeYZ-VpoC complexes conferred resistance to not only bile acids such as cholate and taurocholate but also to some antibiotics and dyes. VmeCD-VpoC conferred high resistance to various drugs. Therefore, complexes with VpoC can extrude various antimicrobial agents more effectively than complexes with TolC.

**Table 4:** Effect of OMPs on MICs for various antimicrobial agents

	MIC (μg mL^−1^)								
	
Antimicrobial agent	KAM43/pBVT3 pSTV28 (control)	KAM43/pBVT3 pRHR229 (*vmeAB*)	KAM43/pBVT3 pSVP201 (*vmeCD*)	KAM43/pBVT3 pSVP202 (*vmeEF*)	KAM43/pBVT3 pSVP204 (*vmeGHI*)	KAM43/pBVT3 pSVP205 (*vmeJK*)	KAM43/pBVT3 pSVP206 (*vmeLM*)	KAM43/pBVT3 pSVP210 (*vmeTUV*)	KAM43/pBVT3 pSVP212 (*vmeYZ*)
Erythromycin	2	16	128	4	2	4	4	2	4
Norfloxacin	0.015	0.125	0.03	0.03	0.015	0.015	0.015	0.015	0.015
Novobiocin	1	32	4	16	1	2	4	1	16
Benzalkonium Cl	4	8	16	4	8	4	4	16	4
Chlorhexidine	2	2	2	2	2	2	2	2	2
Ethidium bromide	2	128	64	32	4	2	2	8	4
Rhodamine 6G	8	128	128	32	8	8	8	16	16
Acriflavine	2	16	4	4	2	2	2	4	2
TPPCl	4	128	128	4	4	4	4	32	8
Crystal violet	1	4	8	1	1	1	1	2	1
Hoechst 33342	0.25	16	1	0.5	0.25	0.5	1	0.5	0.5
SDS	50	800	>51,200	200	800	50	50	400	400
Cholate	2000	8000	16,000	4000	4000	2000	2000	2000	8000
Deoxycholate	125	4000	>32,000	500	500	125	125	500	2000
Taurocholate	2000	>32,000	>32,000	32,000	2000	2000	2000	2000	>32,000
Glycocholate	2000	>32,000	>32,000	8000	2000	2000	2000	2000	>32,000

	MIC (μg mL^−1^)								
	
Antimicrobial agent	KAM43/pBET2 pSTV28 (control)	KAM43/pBET2 pRHR229 (*vmeAB*)	KAM43/pBET2 pSVP201 (*vmeCD*)	KAM43/pBET2 pSVP202 (*vmeEF*)	KAM43/pBET2 pSVP204 (*vmeGHI*)	KAM43/pBET2 pSVP205 (*vmeJK*)	KAM43/pBET2 pSVP206 (*vmeLM*)	KAM43/pBET2 pSVP210 (*vmeTUV*)	KAM43/pBET2 pSVP212 (*vmeYZ*)
Erythromycin	2	16	4	2	2	2	2	2	2
Norfloxacin	0.015	0.125	0.015	0.015	0.015	0.015	0.015	0.015	0.015
Novobiocin	2	128	2	2	2	2	2	2	2
Benzalkonium Cl	4	16	4	4	4	4	4	4	4
Chlorhexidine	2	2	2	2	2	2	2	2	2
Ethidium bromide	2	256	2	2	2	2	2	2	2
Rhodamine 6G	8	128	16	8	8	8	8	8	8
Acriflavine	2	32	2	2	2	2	2	2	2
TPPCl	4	256	16	4	4	4	4	4	4
Crystal violet	1	4	1	1	1	1	1	1	1
Hoechst 33342	0.25	>32	0.25	0.25	0.25	0.25	0.25	0.25	0.25
SDS	100	>51,200	>51,200	50	100	50	50	50	>51,200
Cholate	4000	>32,000	>32,000	4000	4000	4000	4000	4000	16,000
Deoxycholate	500	>32,000	>32,000	500	500	500	500	500	1000
Taurocholate	4000	>32,000	>32,000	4000	4000	4000	4000	4000	>32,000
Glycocholate	4000	>32,000	>32,000	4000	4000	4000	4000	4000	>32,000

VpoC was expressed from the multicopy plasmid while TolC was expressed from the chromosome. Therefore, the expression of outer membrane components may affect the activity of RND-type efflux transporters. We measured MICs of several drugs to KAM43 harboring pBET2, carrying the *tolC* of *E. coli*, and the recombinant plasmid carrying each RND-type efflux transporter gene of *V. parahaemolyticus*. However, the MICs of KAM43/pBET2 harboring each recombinant plasmid were similar to those of KAM33 harboring each recombinant plasmid (Table 4, lower panel). Therefore, we concluded that the RND-type efflux transporters of *V. parahaemolyticus* could utilize VpoC better than TolC.

**Figure 2:**
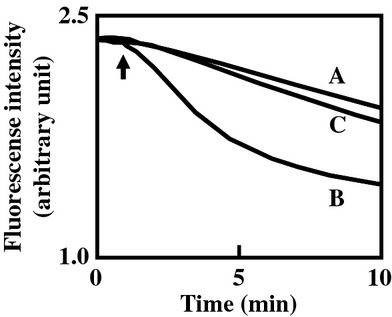
Active efflux of ethidium from *E. coli* cells expressing VmeCD-VpoC or VmeEF-VpoC. Energy-starved *E. coli* cells were loaded with 10 μmol/L ethidium. At the time point indicated by the arrow, potassium lactate (final concentration, 40 mmol/L) was added to energize cells. Ethidium efflux was represented by a rapid decrease in fluorescence. (A) KAM43/pSTV28/pBVT3 (*vpoC* only); (B) KAM43/pSVP201/pBVT3 (*vmeCD+vpoC*); (C) KAM43/pSVP202/pBVT3 (*vmeEF+vpoC*).

We measured ethidium efflux via VmeCD-VpoC and VmeEF-VpoC in *E. coli* cells. The fluorescence of ethidium has been shown to increase when it binds to DNA inside the cell. Therefore, the efflux of ethidium can be detected as a decrease in fluorescence intensity (LePecq and Paoletti 1967). After the addition of potassium lactate to energy-starved and ethidium-preloaded cells, *E. coli* expressing VmeCD-VpoC had significantly lower intracellular ethidium levels than those of the control cells expressing only VpoC (Fig. 2). In the case of *E. coli* expressing VmeEF-VpoC, intracellular ethidium levels were slightly but reproducibly decreased. These results suggest that VmeCD-VpoC and VmeEF-VpoC are energy-dependent drug efflux transporters.

### RT-PCR analysis of the RND-type efflux transporter genes

**Figure 3:**
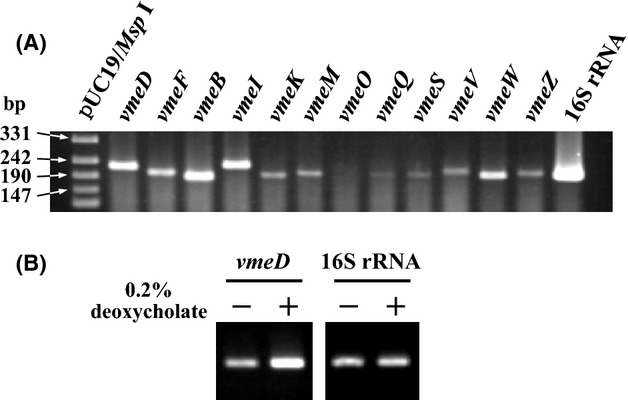
RT–PCR analyses in *V. parahaemolyticus* AQ3334. (A) Total RNA (0.5 μg) purified from exponential-phase-grown cells was used for RT-PCR. Amplifications for all RND-type efflux transporter genes and 16S rRNA were performed in 23 cycles. (B) Cells were grown until the exponential phase in the presence or absence of 0.2% sodium deoxycholate, (+) and (−), respectively. One nanogram of total RNA was used for RT-PCR. Amplifications for *vmeD* and 16S rRNA were performed in 28 cycles or 13 cycles, respectively. The absence of DNA contamination was confirmed using total RNA without reverse transcription as a template.

We investigated the expression of 12 RND-type efflux transporter genes in *V. parahaemolyticus* AQ3334 using the RT-PCR method. The expression of *vmeB*, *vmeD*, *vmeI,* and *vmeW* was relatively abundant among the 12 RND-type efflux transporter genes (Fig. 3A). In contrast, the expression of *vmeO*, *vmeQ,* and *vmeS* was low. The expression pattern of RND-type efflux transporter genes in *V. parahaemolyticus* RIMD2210633 was similar to that in AQ3334 (data not shown).

The expression of both *vexAB* and *vexCD*, which encode RND-type efflux transporters, in *V. cholerae* was shown to be upregulated in response to bile acids (Bina et al. 2006). We tested whether the expression of the 12 RND-type efflux transporter genes was influenced by bile acids. The RT-PCR results showed that the expression of *vmeD* was upregulated in response to deoxycholate, which is one of the constituents of bile acids (Fig. 3B). The putative TetR family transcriptional regulator gene, VP0040, was located upstream of *vmeC*. This regulator protein encoded by VP0040 may be involved in the transcriptional regulation of *vmeCD*. The transcription of other RND-type efflux transporter genes was not affected by deoxycholate (data not shown).

### Role of RND-type efflux transporters in intrinsic resistance to various antimicrobial agents in *V. parahaemolyticus*

We previously showed that VmeAB conferred high resistance to various drugs and bile acids on *E. coli* KAM33 cells. However TM3, which is the *vmeAB* deletion mutant from *V. parahaemolyticus* AQ3334, had slightly lower resistance levels to various drugs than that of AQ3334 (Matsuo et al. 2007). To investigate the contributions of other RND-type efflux transporters to the intrinsic resistance of *V. parahaemolyticus*, we constructed deletion mutants of the *V. parahaemolyticus* AQ3334 strain lacking the RND-type efflux transporter genes (Table 1). TM4 (*ΔvmeCD*), in addition to TM3 (*ΔvmeAB*), had slightly increased susceptibility to the several antimicrobial agents tested. However, other single deletion mutants showed the same susceptibility as AQ3334 (Table 5).

**Table 5:** Susceptibility of *V. parahaemolyticus* RND-type efflux transporter deficient mutants to various antimicrobial agents

	MIC (μg mL^−1^)																			
	
Strain	Deleted genes^1^	OXA	CLO	EM	NFLX	NOV	TET	KM	CM	BC	CHL	EtBr	R6G	ACR	TPP	HOE	SDS	CHO	DEO	GLY
AQ3334	parental strain	128	128	4	0.06	8	1	16	0.5	16	32	128	128	16	128	2	51,200	>32,000	8000	>32,000
TM3	AB	**64**	128	4	0.06	8	1	16	0.5	16	32	**64**	128	**8**	128	**0.5**	51,200	>32,000	8000	>32,000
TM4	CD	**64**	**32**	**2**	0.06	8	1	16	0.5	16	**16**	128	**16**	16	**64**	2	**1600**	**4000**	**2000**	>32,000
TM5	EF	128	128	4	0.06	8	1	16	0.5	16	32	128	128	16	128	2	51,200	>32,000	8000	>32,000
TM6	HI	128	128	4	0.06	8	1	16	0.5	16	32	128	128	16	128	2	51,200	>32,000	8000	>32,000
TM7	K	128	128	4	0.06	8	1	16	0.5	16	32	128	128	16	128	2	51,200	>32,000	8000	>32,000
TM8	LM	128	128	4	0.06	8	1	16	0.5	16	32	128	128	16	128	2	51,200	>32,000	8000	>32,000
TM9	O	128	128	4	0.06	8	1	16	0.5	16	32	128	128	16	128	2	51,200	>32,000	8000	>32,000
TM10	Q	128	128	4	0.06	8	1	16	0.5	16	32	128	128	16	128	2	51,200	>32,000	8000	>32,000
TM11	RS	128	128	4	0.06	8	1	16	0.5	16	32	128	128	16	128	2	51,200	>32,000	8000	>32,000
TM12	UV	128	128	4	0.06	8	1	16	0.5	16	32	128	128	16	128	2	51,200	>32,000	8000	>32,000
TM13	WX	128	128	4	0.06	8	1	16	0.5	16	32	128	128	16	128	2	51,200	>32,000	8000	>32,000
TM14	YZ	128	128	4	0.06	8	1	16	0.5	16	32	128	128	16	128	2	51,200	>32,000	8000	>32,000
TM32	AB, CD	**32**	**8**	**0.25**	**0.03**	**2**	1	16	0.5	**8**	**16**	**8**	**8**	**8**	**16**	**0.5**	**400**	**2000**	**1000**	>32,000
TM33	AB, CD, EF	**16**	**8**	**0.25**	**0.03**	**0.5**	1	16	0.5	**8**	**16**	**4**	**8**	**8**	**16**	**0.5**	**200**	**1000**	**1000**	>32,000
TM34	AB, CD, EF, YZ	**16**	**2**	**0.25**	**0.03**	**0.5**	1	16	0.5	**8**	**8**	**4**	**8**	**8**	**16**	**0.5**	**200**	**1000**	**1000**	**1000**
TM35	AB, CD, EF, YZ, K	**16**	**2**	**0.25**	**0.03**	**0.25**	1	16	0.5	**8**	**8**	**4**	**8**	**8**	**16**	**0.5**	**200**	**1000**	**1000**	**1000**
TM36	AB, CD, EF, YZ, K, HI	**16**	**2**	**0.25**	**0.03**	**0.25**	1	16	0.5	**8**	**8**	**4**	**8**	**8**	**16**	**0.5**	**100**	**1000**	**500**	**1000**
TM37	AB, CD, EF, YZ, K, HI, LM	**16**	**2**	**0.25**	**0.03**	**0.25**	1	16	0.5	**4**	**4**	**4**	**2**	**8**	**16**	**0.5**	**50**	**500**	**125**	**1000**
TM38	AB, CD, EF, YZ, K, HI, LM, UV	**16**	**2**	**0.25**	**0.03**	**0.25**	1	16	0.5	**4**	**4**	**4**	**2**	**8**	**16**	**0.5**	**50**	**500**	**125**	**1000**
TM39	AB, CD, EF, YZ, K, HI, LM, UV, WX	**16**	**2**	**0.25**	**0.03**	**0.25**	1	16	0.5	**4**	**4**	**4**	**2**	**8**	**16**	**0.5**	**50**	**500**	**125**	**1000**
TM310	AB, CD, EF, YZ, K, HI, LM, UV, WX, Q	**16**	**2**	**0.25**	**0.03**	**0.25**	1	16	0.5	**4**	**4**	**4**	**2**	**8**	**16**	**0.5**	**50**	**500**	**125**	**1000**
TM311	AB, CD, EF, YZ, K, HI, LM, UV, WX, Q, RS	**16**	**2**	**0.25**	**0.03**	**0.25**	1	16	0.5	**4**	**4**	**4**	**2**	**8**	**16**	**0.5**	**50**	**500**	**125**	**1000**
TM312	AB, CD, EF, YZ, K, HI, LM, UV, WX, Q, RS, O	**16**	**2**	**0.25**	**0.03**	**0.25**	1	16	0.5	**4**	**4**	**4**	**2**	**8**	**16**	**0.5**	**50**	**500**	**125**	**1000**
TM15	*vpoC*	**4**	**1**	**0.25**	**0.03**	**0.25**	1	16	0.5	**4**	**4**	**4**	**2**	**8**	**16**	**0.5**	**50**	**500**	**125**	**1000**
TM425	AB, CD, EF, YZ, K, HI, LM, UV, WX, Q, RS, O, *vpoC*	**4**	**1**	**0.25**	**0.03**	**0.25**	1	16	0.5	**4**	**4**	**4**	**2**	**8**	**16**	**0.5**	**50**	**500**	**125**	**1000**

OXA, oxacillin; CLO, cloxacillin; EM, erythromycin; NFLX, norfloxacin; NOV, novobiocin; TET, tetracycline; KM, kanamycin; CM, chloramphenicol; BC, benzalkonium chloride; CHL, chlorhexidine; EtBr, ethidium bromide; R6G, rhodamine 6G; ACR, acriflavine; TPP; tetraphenylphosphonium chloride; HOE, Hoechst 33342; SDS, sodium dodecyl sulfate; CHO, sodium cholate; DEO, sodium deoxycholate; GLY, sodium glycocholate. MIC values in boldface are smaller than those of the parent strain *V. parahaemolyticus* AQ3334.

1 Deleted genes: ‘*vme*’ was omitted.

Most single deletions had no effect on drug susceptibility even if some deleted genes conferred drug resistance when expressed in *E. coli* cells. The effect of a single gene deletion may be masked by other efflux systems. Therefore, we constructed multiple gene disruption strains (Table 1). We also constructed TM312, in which all of the 12 RND-type efflux transporter genes are deleted, and investigated the drug susceptibilities of these mutants (Table 5). We investigated growth rates before any of the experiments were conducted. Under our experimental conditions, all of the deletion strains showed comparative growth rates.

Double disruptant TM32 (*ΔvmeAB*, *ΔvmeCD*) showed higher susceptibility than TM3 to many antimicrobial agents, such as cloxacillin (16-fold), erythromycin (16-fold), novobiocin (fourfold), ethidium bromide (eightfold), rhodamine 6G (16-fold), tetraphenylphosphonium chloride (TPPCl) (fourfold), sodium dodecyl sulfate (SDS) (128-fold), sodium cholate (CHO) (more than 16-fold), and sodium deoxycholate (DEO) (eightfold). The triple deletion strain TM33 with *vmeEF* deleted was more susceptible than parental TM32 to oxacillin (twofold), novobiocin (fourfold), ethidium bromide (twofold), SDS (twofold), and CHO (twofold). TM34 with *vmeYZ* deleted was more susceptible than TM33 to cloxacillin (fourfold), chlorhexidine (twofold), and sodium glycocholate (GLY) (more than 32-fold). Moreover, TM37 with *vmeJK*, *vmeGHI*, and *vmeTUV* deleted was more susceptible to benzalkonium chloride (twofold), chlorhexidine (twofold), rhodamine 6G (fourfold), SDS (fourfold), CHO (twofold), and DEO (eightfold). Deletion of the remaining RND-type efflux transporter genes (*vmeLM*, *vmeNO*, *vmePQ*, *vmeRS,* and *vmeWX*) from TM37 did not increase susceptibility to any of the antimicrobial agents tested. These results suggest that at least seven transporters (VmeAB, VmeCD, VmeEF, VmeYZ, VmeJK, VmeGHI, and VmeTUV) are functional and contribute to intrinsic resistance in *V. parahaemolyticus* against several antimicrobial agents.

We also constructed the *vpoC* deletion mutant TM15 from *V. parahaemolyticus* AQ3334. TM15 showed similar substrate specificity profiles to TM312, which suggests that the RND-type efflux transporters that are involved in the intrinsic resistance of *V. parahaemolyticus* require VpoC as an OMP component. Furthermore, TM15 had higher susceptibility to oxacillin and cloxacillin than that of TM312, which indicates that efflux systems, except the RND-type efflux transporter, exist in *V. parahaemolyticus* and interact with VpoC.

**Figure 4:**
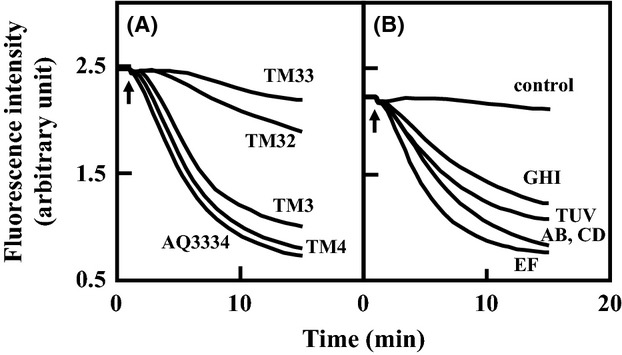
Active efflux of ethidium from *V. parahaemolyticus* cells. At the time point indicated by the arrow, potassium lactate (final concentration, 40 mmol/L) was added to energize cells. (A) Active efflux in a series of gene disruptants. (B) Active efflux in TM312 expressing indicated RND-type transporters. TM312 carrying pSTV28 (empty vector) was used as a control.

We measured the efflux of ethidium in *V. parahaemolyticus* AQ3334 and its derivatives. *V. parahaemolyticus* AQ3334 exhibited strong efflux after the addition of potassium lactate (Fig. 4A). TM3 and TM4, lacking either *vmeAB* or *vmeCD*, showed similar effluxes to the parental strain AQ3334. However, marked decreases in ethidium efflux were observed in TM32 lacking both *vmeAB* and *vmeCD*. Efflux was less by the triple disruptant TM33 than by TM32. TM34 and TM312 showed similar effluxes to TM33 (data not shown). These results suggest that a deficiency in one efflux transporter is complemented by another efflux transporter, which corresponds to the results of the drug susceptibility test.

### Characterization of RND-type efflux transporters in V. parahaemolyticus cells

To characterize each RND-type efflux transporter in *V. parahaemolyticus*, we introduced recombinant plasmids carrying each RND-type efflux transporter gene into *V. parahaemolyticus* TM312, and investigated drug susceptibility (Table 6). The introduction of pRHR228 (*vmeAB*) and pSVP201 (*vmeCD*) conferred drug resistance to many antimicrobial agents on *V. parahaemolyticus* TM312 cells, and its substrate profiles were similar to those of *E. coli* cells (Table 3). The introduction of either VmeAB or VmeCD by itself could restore drug resistance to wild type levels. Interestingly, some RND-type efflux transporters exhibited substrate specificity profiles in *V. parahaemolyticus* cells that differ from those in *E. coli* cells. pSVP205 (*vmeJK*) and pSVP206 (*vmeLM*) were shown to newly confer drug resistance to rhodamine 6G and bile acids, respectively. Although none of the RND type efflux transporters exhibited elevated resistance to chlorhexidine in *E. coli* cells, VmeAB, VmeCD, VmeGHI, and VmeTUV conferred drug resistance to chlorhexidine in *V. parahaemolyticus* cells. Furthermore, pSVP208 (*vmePQ*) conferred drug resistance to SDS and bile acids on *V. parahaemolyticus* cells, whereas the plasmid did not confer any resistance on *E. coli* cells.

**Table 6:** Susceptibility of *V. parahaemolyticus* TM312-overproduced RND-type efflux transporters to various antimicrobial agents

	MIC (μg mL^−1^)																	
	
Strain	OXA	CLO	EM	NFLX	NOV	TET	KM	BC	CHL	EtBr	R6G	ACR	TPP	HOE	SDS	CHO	DEO	GLY
AQ3334	128	128	4	0.06	8	1	16	16	32	128	128	16	128	2	51,200	>32,000	8000	>32,000
TM312/pSTV28	16	2	0.25	0.03	0.25	1	16	4	4	4	2	8	16	0.5	50	500	125	1000
TM312/pRHR228 (AB)	**128**	**128**	**1**	**0.06**	**8**	1	16	**16**	**32**	**128**	**64**	**32**	**128**	**4**	**25,600**	**16,000**	**4000**	**>32,000**
TM312/pSVP201 (CD)	**128**	**256**	**16**	0.03	**1**	1	16	**32**	**64**	**128**	**256**	**16**	**256**	0.5	**51,200**	**>32,000**	**16,000**	**>32,000**
TM312/pSVP202 (EF)	16	**8**	**0.5**	0.03	**1**	1	16	4	4	**32**	**8**	**16**	16	0.5	**200**	**2000**	**500**	**>32,000**
TM312/pSVP204 (GHI)	16	2	0.25	0.03	0.25	1	16	**32**	**8**	**8**	2	8	16	0.5	**1600**	**2000**	**1000**	**>32,000**
TM312/pSVP205 (JK)	16	2	**1**	0.03	0.25	1	16	4	4	**8**	**32**	8	16	0.5	50	500	125	1000
TM312/pSVP206 (LM)	16	**4**	**1**	0.03	**0.5**	1	16	4	4	4	2	8	16	**2**	50	**1000**	**250**	**4000**
TM312/pSVP207 (NO)	16	2	0.25	0.03	0.25	1	16	4	4	4	2	8	16	0.5	50	500	125	1000
TM312/pSVP208 (PQ)	16	**4**	0.25	0.03	0.25	1	16	4	4	4	2	8	16	0.5	**100**	**1000**	**500**	**8000**
TM312/pSVP209 (RS)	16	2	0.25	0.03	0.25	1	16	4	4	4	2	8	16	0.5	50	500	125	1000
TM312/pSVP210 (TUV)	16	**32**	0.25	0.03	0.25	1	16	**32**	**32**	**64**	**32**	**64**	**64**	0.5	**800**	**4000**	**2000**	**>32,000**
TM312/pSVP211 (WX)	16	2	0.25	0.03	0.25	1	16	4	4	4	2	8	16	0.5	50	500	125	1000
TM312/pSVP212 (YZ)	**64**	**64**	**0.5**	0.03	**1**	1	16	**8**	4	**8**	**8**	8	16	0.5	**25,600**	**8000**	**4000**	**>32,000**

OXA, oxacillin; CLO, cloxacillin; EM, erythromycin; NFLX, norfloxacin; NOV, novobiocin; TET, tetracycline; KM, kanamycin; BC, benzalkonium chloride; CHL, chlorhexidine; EtBr, ethidium bromide; R6G, rhodamine 6G; ACR, acriflavine; TPP; tetraphenylphosphonium chloride; HOE, Hoechst 33342; SDS, sodium dodecyl sulfate; CHO, sodium cholate; DEO, sodium deoxycholate; GLY, sodium glycocholate. MIC values in boldface are larger than those of the control strain.

Previous studies showed that MexJK required OprM and OpmH as OMP components for erythromycin efflux and triclosan efflux in *P. aeruginosa*, respectively (Chuanchuen et al. 2002, 2005). Therefore, we attempted to identify any OMP component, except for VpoC, required in *V. parahaemolyticus*. Recombinant plasmids carrying each RND transporter gene were introduced into TM425, which is the deleted *vpoC* gene from TM312. No RND-type efflux pumps elevated resistance to any antimicrobial agent (data not shown). Nine RND-type efflux transporters, which conferred drug resistance on *V. parahaemolyticus* TM312, were suggested to utilize only VpoC as an OMP component.

We also measured the efflux of ethidium in *V. parahaemolyticus* TM312 cells harboring pRHR228 (*vmeAB*), pSVP201 (*vmeCD*), pSVP202 (*vmeEF*), pSVP204 (*vmeGHI*), and pSVP210 (*vmeTUV*), which conferred ethidium resistance on TM312. As shown in Figure 4B, the energy-dependent efflux of ethidium from *V. parahaemolyticus* TM312 cells harboring each plasmid was observed as a decrease in fluorescence. Because the control cells of TM312/pSTV28 showed very low ethidium efflux activity, VmeAB, VmeCD, VmeEF, VmeGHI, and VmeTUV were suggested to possess the efflux ability of ethidium.

### Physiological properties of RND-type efflux transporter disruptants

The *ΔvmeAB* strain showed slight sensitivity to deoxycholate; therefore, VmeAB was considered to contribute to resistance to deoxycholate (Matsuo et al. 2007). The MIC for deoxycholate revealed that at least eight RND-type efflux transporters conferred resistance to sodium deoxycholate. We next investigated the effect of sodium deoxycholate on *V. parahaemolyticus* gene deletion mutants. The percentage of viable AQ3334 cells exposed to 1 mg mL^−1^ sodium deoxycholate was reduced to 16%. In contrast, TM3 (*ΔvmeAB*) and TM4 (*ΔvmeCD*) showed 83% and 99.9% reductions in survival rates, respectively (Table 7). Viable cells of the TM32 and TM312 strains treated with deoxycholate were not detected in our experimental conditions. These results suggest that VmeCD is the main efflux transporter with roles in the resistance to deoxycholate, with VmeAB being the next contributor.

**Table 7:** Effect of sodium deoxycholate on the survival of *V. parahaemolyticus* cells

Strain	Survival rate (%)
AQ3334	84
TM3	17
TM4	0.070
TM32	Undetec*
TM312	Undetec*

Three individual experiments were performed and similar results were obtained. Representative results were shown.

* Undetectable: the detection limit of this experiment was 1.64 × 10^−4^%.

**Figure 5:**
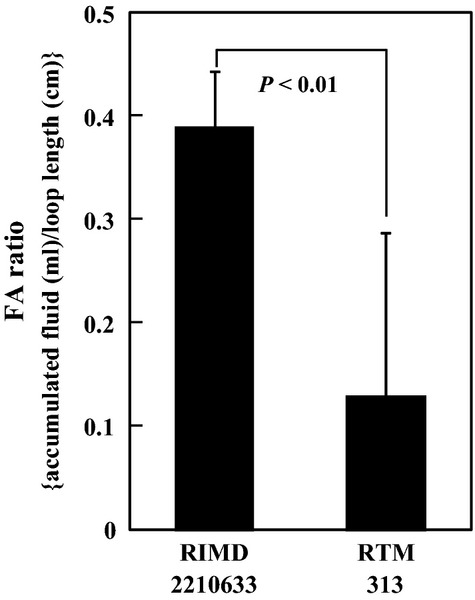
Involvement of RND-type efflux transporters in induced enterotoxicity. The enterotoxicity of the 12 RND-type efflux transporter gene-deficient strain, RTM313, was evaluated by the rabbit ileal loop test. The fluid accumulation (FA) ratio in each loop was measured 18 h after the injection. FA was measured by the amount of accumulated fluid (in mL) per length (in cm) of ligated rabbit small intestine. Error bars represent standard deviations. Three independent experiments were performed and a significant difference was observed (*P* < 0.005).

Fluid accumulation was compared in rabbit ileal loops to evaluate the contribution of RND-type efflux transporters to its pathogenicity. The *V. parahaemolyticus* RIMD2210633 strain was used in this experiment because this strain was shown to have the ability to cause fluid accumulation (Gotoh et al. 2010). All 12 RND-type efflux transporter genes were deleted from RIMD2210633, and it was subsequently designated as RTM313. The MIC values of RTM313 were almost the same as those of TM312 (data not shown). Fluid accumulation was significantly lower with RTM313 than with RIMD2210633 (Fig. 5), which indicates that RND-type efflux transporters are involved in the pathogenicity of *V. parahaemolyticus*.

**Figure 6:**
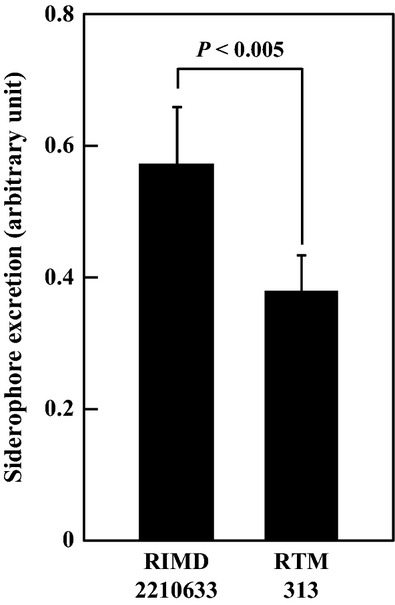
Excretion of siderophores from *V. parahaemolyticus* cells. Excreted siderophores from *V. parahaemolyticus* cells were detected with the CAS assay. An aliquot of the supernatant of the culture was mixed with the CAS assay solution and was incubated at room temperature for 2 h. Siderophore excretion was measured by the absorbance of the reaction mixture at 630 nm per O.D._650_ when cells were harvested.

We next investigated the production of siderophores, which bind ferric ions and are important for the uptake of iron. MexAB was shown to be involved in the secretion of pyoverdine, a siderophore in *P. aeruginosa*, and *V. parahaemolyticus* was also shown to produce the polyhydroxycarboxylate-type siderophore, vibrioferrin under iron-limiting conditions (Yamamoto et al. 1994). Vibrioferrin production was higher by the *V. parahaemolyticus* RIMD2210633 strain than by the AQ3334 strain (data not shown). Therefore, RIMD2210633 and its derivatives were used to investigate vibrioferrin production. The CAS assay revealed that extracellular vibrioferrin was significantly decreased in RTM313 (Fig. 6), which suggests that some RND-type efflux transporters are important for vibrioferrin export or production.

We also investigated cell morphology and biofilm formation. No significant morphological changes were observed between TM312 and wild type cells (data not shown). We used *V. parahaemolyticus* RIMD2210633 and its derivatives for the biofilm formation assay because *V. parahaemolyticus* AQ3334 could not form biofilms under our experimental conditions (data not shown). The deletion of any RND-type efflux transporter had no significant effect on biofilm formation (data not shown).

## Discussion

We revealed that there are 12 operons on the chromosomal DNA of *V. parahaemolyticus* RIMD2210633, and, judging from sequence similarities, these operons code for putative RND-type efflux transporters. Among them, we showed that eight transporters including VmeAB (Matsuo et al. 2007) had the ability to confer resistance to host *E. coli* cells. All RND-type efflux transporters estimated from genome sequences have been characterized in three bacteria. All 11 transporters were shown to have the ability to exclude several antimicrobial agents in *P. aeruginosa* (Poole et al. 1993, 1996; Kohler et al. 1997; Mine et al. 1999; Westbrock-Wadman et al. 1999; Aendekerk et al. 2002; Chuanchuen et al. 2002; Li et al. 2003; Sekiya et al. 2003; Mima et al. 2005, 2007), while some RND-type transporters could function as efflux transporters in *E. coli* and *V. cholerae* (Nishino and Yamaguchi 2001; Rahman et al. 2007). These studies and present findings suggest that bacterial cells contain ‘multiple’ and ‘active’ RND-type efflux transporters, which appear to emphasize the importance of this type of transporter. We cannot exclude the possibility that the remaining three RND-type efflux transporters (VmeNO-VpoM, VmeRS, and VmeWX) are functional. Other possibilities need to be verified including the existence of other substrates to those tested in this study and the necessity of any other subunits. Furthermore, a more effective protein expression system should be attempted for *V. parahaemolyticus*.

In this study, we found several important physiological roles for RND-type transporters. Multiple deletions of these genes significantly increased the sensitivity of *V. parahaemolyticus* to many antimicrobial agents including deoxycholate. In particular, the MICs of all 12 deletion strains were markedly reduced and fluid accumulation in rabbit ileal loops was also markedly decreased. Although it remains to be elucidated whether this change was due to RND transporters influencing diarrheic activity in rabbit ileal loops or simply that the strain could not survive in the intestine, these results revealed that RND-type transporters play very important roles in the intrinsic resistance of *V. parahaemolyticus*. In addition, the sensitivity of the double deletion strain TM32 (*ΔvmeAB* and *ΔvmeCD*) to deoxycholate exposure and the induction of *vmeCD* expression in the presence of deoxycholate suggest that VmeCD could be the main RND-type multidrug efflux transporter followed by VmeAB.

We investigated the expression of 12 RND-type efflux transporter genes in *V. parahaemolyticus*. The mRNAs of 11 genes were detected in varying degrees. This expression profile was observed when cells were cultured in rich medium under laboratory conditions. However, the expression of RND-type efflux transporters was shown to be controlled with several transcriptional regulators (Grkovic et al. 2002). *vmeD* was highly expressed in the presence of deoxycholate (Fig. 3); therefore, the expression of other genes may be induced when cells are in the intestine or are exposed to antimicrobial agents. More analyses should be performed to elucidate these complex regulatory mechanisms.

**Figure 7:**
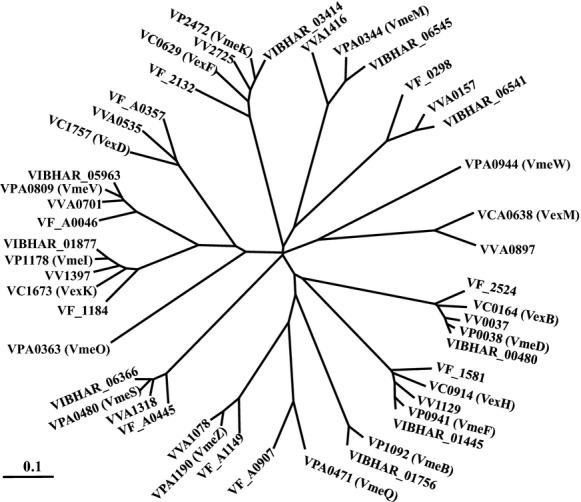
Phylogenetic tree for the RND family of multidrug transporters. The phylogenetic tree was obtained using the CLUSTAL W system provided by the DNA Data Bank of Japan (available at the website http://clustalw.ddbj.nig.ac.jp/top-j.html). The amino acid sequences of IMP components for RND-type efflux pumps of *V. parahaemolyticus*, *V. cholerae*, *V. fischeri*, *V. harveyi,* and *V. vulnificus* were applied to the CLUSTAL W system.

When compared with already characterized RND transporters, it is reasonable to assume that the primary amino acid sequence and characteristics of efflux transporters are tightly associated. In Figure 7, phylogenetic analyses of IMP components for RND-type efflux transporters in *V. parahaemolyticus*, *V. cholerae*, *V. fischeri*, *V. harveyi,* and *V. vulnificus* revealed that VmeD may be an orthologue of VexB in *V. cholerae* (Bina et al. 2006; Rahman et al. 2007). The substrate specificity of VmeCD was similar to that of VexAB. The ability of VmeCD to elevate MICs and export intracellular ethidium was significantly high (Table 6 and Fig. 4). Moreover, the putative orthologues of VmeCD are present in all five *Vibrionaceae*. These facts remind us that VmeCD and its orthologs are one of the main multidrug efflux transporters not only in *V. parahaemolyticus* but also in *Vibrionaceae*. The lack of VexAB in *V. cholerae*, which is the orthologue of VmeCD, conferred sensitivity to several antimicrobial reagents (Bina et al. 2006). Therefore, VmeCD could be the main and most common RND-type efflux transporter in *Vibrionaceae*.

On the other hand, VmeAB appears to be an atypical transporter. Although the ability of VmeAB to exclude several substrates was higher than the other transporters, its orthologue has only been reported in *V. harveyi*. We previously demonstrated that VmeAB was more similar to the AcrAB of *E. coli* than to any other RND-type efflux transporter in *Vibrionaceae* (Matsuo et al. 2007). On the basis of our present results, we focused on the ability of the transporters to utilize TolC. Of the 12 RND-type efflux transporters studied, only VmeAB could utilize TolC as well as VpoC, while the others preferred VpoC to TolC. Therefore, it is possible that the origin of VmeAB is different from the other vibrio RND transporters, and *V. parahaemolyticus* may have acquired *vmeAB* elsewhere after dividing from the other *Vibrionaceae* during evolution.

Four RND-type efflux transporters (VmeAB, VmeCD, VmeEF, and VmeYZ) out of 12 could utilize TolC as an OMP component and conferred drug resistance on *E. coli* KAM33 cells. However, when the RND-type efflux transporters of *V. parahaemolyticus* functioned with VpoC as the outer membrane component in *E. coli* KAM43 cells, eight (VmeAB, VmeCD, VmeEF, VmeGHI, VmeJK, VmeLM, VmeTUV, and VmeYZ) of the transporters conferred drug resistance. All of them, except for VmeAB exhibited broader substrate specificity and conferred higher drug resistance than when functioning with TolC. Three possibilities have been suggested for this phenomenon: (i) VmeGHI, VmeJK, VmeLM, and VmeTUV cannot form functional tripartite complexes with TolC, but can interact with VpoC, which is a putative primary OMP of *V. parahaemolyticus*, (ii) all substrates are extruded by both complexes with TolC and the complexes with VpoC, while effluxes via complexes with TolC are much lower; consequently, we cannot detect differences as changes in MIC values, and (iii) complexes with VpoC can recognize some drugs as substrates that complexes with TolC cannot. At present, it is difficult to judge which possibilities are correct.

Nine RND-type efflux transporters that conferred elevated resistance utilized VpoC as their OMPs (Table 4). The relationships between IMP, MFP, and OMP have been reported in several bacteria. TolC in *E. coli* is coordinated with AcrAB, AcrD (with AcrA), AcrEF, MdtABC, and MdtEF (Nishino et al. 2003). In *Salmonella* Typhimurium, AcrAB, AcrD (with AcrA), AcrEF, MdsAB, and MdtABC were shown to be dependent on TolC (Horiyama et al. 2010). However, in the case of *P. aeruginosa*, certain RND-type efflux transporters utilize multiple OMPs (Gotoh et al. 1998; Yoneyama et al. 1998; Maseda et al. 2000; Murata et al. 2002). The VpoC deletion strain TM15 showed hypersensitivity to many antimicrobial agents, relative to TM312, which indicated that VpoC may play a central role as an OMP in *V. parahaemolyticus* such as *E. coli* and *S*. Typhimurium.

We finally constructed a mutant in which all 12 RND efflux transporter genes were deleted. In this study, we showed that the roles of RND efflux transporters include resistance to deoxycholate and antimicrobial agents, pathogenicity in the intestine, and the export of siderophores. However, our experimental growth condition is ‘overprotective’. Other functions may be found in more severe conditions, such as in sea water or in the midgut gland of bivalves. The mutant series that was generated in this study can contribute to the identification of new roles for RND-type efflux transporters in *Vibrionaceae*.
